# Inflammatory response in traumatic brain and spinal cord injury: The role of XCL1‐XCR1 axis and T cells

**DOI:** 10.1111/cns.14781

**Published:** 2024-06-17

**Authors:** Mingkang Zhang, Xiaonan Han, Liyan Yan, Yikun Fu, Hongwei Kou, Chunfeng Shang, Junmin Wang, Hongjian Liu, Chao Jiang, Jian Wang, Tian Cheng

**Affiliations:** ^1^ Department of Orthopaedics The First Affiliated Hospital of Zhengzhou University Zhengzhou Henan China; ^2^ Department of Human Anatomy, School of Basic Medical Sciences Zhengzhou University Zhengzhou Henan China; ^3^ Department of Neurology People's Hospital of Zhengzhou University Zhengzhou Henan China

**Keywords:** inflammatory response, spinal cord injury, T‐cell response, traumatic brain injury, XCL1‐XCR1 axis

## Abstract

**Background:**

Traumatic brain injury (TBI) and spinal cord injury (SCI) are acquired injuries to the central nervous system (CNS) caused by external forces that cause temporary or permanent sensory and motor impairments and the potential for long‐term disability or even death. These conditions currently lack effective treatments and impose substantial physical, social, and economic burdens on millions of people and families worldwide. TBI and SCI involve intricate pathological mechanisms, and the inflammatory response contributes significantly to secondary injury in TBI and SCI. It plays a crucial role in prolonging the post‐CNS trauma period and becomes a focal point for a potential therapeutic intervention. Previous research on the inflammatory response has traditionally concentrated on glial cells, such as astrocytes and microglia. However, increasing evidence highlights the crucial involvement of lymphocytes in the inflammatory response to CNS injury, particularly CD8^+^ T cells and NK cells, along with their downstream XCL1‐XCR1 axis.

**Objective:**

This review aims to provide an overview of the role of the XCL1‐XCR1 axis and the T‐cell response in inflammation caused by TBI and SCI and identify potential targets for therapy.

**Methods:**

We conducted a comprehensive search of PubMed and Web of Science using relevant keywords related to the XCL1‐XCR1 axis, T‐cell response, TBI, and SCI.

**Results:**

This study examines the upstream and downstream pathways involved in inflammation caused by TBI and SCI, including interleukin‐15 (IL‐15), interleukin‐12 (IL‐12), CD8^+^ T cells, CD4^+^ T cells, NK cells, XCL1, XCR1^+^ dendritic cells, interferon‐gamma (IFN‐γ), helper T0 cells (Th0 cells), helper T1 cells (Th1 cells), and helper T17 cells (Th17 cells). We describe their proinflammatory effect in TBI and SCI.

**Conclusions:**

The findings suggest that the XCL1‐XCR1 axis and the T‐cell response have great potential for preclinical investigations and treatments for TBI and SCI.

## Graphical abstract

The role of XCL1‐XCR1 axis and T cells in traumatic brain and spinal cord injury.

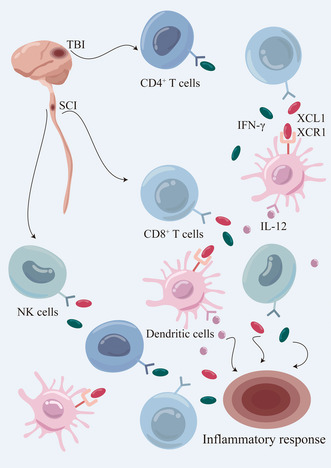

## 1 INTRODUCTION

The central nervous system (CNS) is a complex network comprising the brain and the spinal cord, which regulates bodily functions through a complex network of neuronal pathways and interconnections.^1^ However, CNS injuries can lead to two prevalent and challenging conditions, traumatic brain injury (TBI) and spinal cord injury (SCI), both of which have high incidence rates and limited recovery rates.^2^, ^3^ Globally, TBI affects over 50 million people annually,^3^, ^4^ while more than 27 million people worldwide experience long‐term disabilities due to SCI.^5^, ^6^

The current medical interventions available for TBI and SCI do not provide effective treatments for these conditions. This results in patients with persistent deficits in sensory, motor, and autonomic functions,^7^, ^8^ as well as dealing with persistent pain, impaired mobility, and the burden of chronic anxiety and depression.^9^, ^10^ Furthermore, TBI and SCI impose a substantial economic burden on patients and healthcare systems worldwide, in addition to patients' profound physical constraints.^11^, ^12^ Therefore, it is imperative to develop practical and effective interventions to alleviate the profound suffering experienced by patients.^12^, ^13^, ^14^ The pressing need for such interventions is due to the high incidence and limited recovery rates associated with these conditions and the substantial economic burden they impose.

TBI and SCI exhibit several commonalities that require a comprehensive discussion to foster the exchange of relevant research and insights. The injuries associated with both conditions can be categorized as primary and secondary. Primary injuries alter the integrity of the blood‐spinal cord or the blood–brain barrier,^8^, ^15^, ^16^ creating a favorable environment for infiltrating various inflammatory cells. Secondary injuries are the result of inflammatory responses, ischemia, edema, and oxidative stress. Of note, the inflammatory response is crucial in causing secondary damage to the nervous system, making it a vital focus in treating TBI and SCI.^17^, ^18^ Previous research has consistently underscored the importance of microglia and astrocytes in orchestrating the inflammatory response after CNS injury.^3^, ^19^, ^20^ These specialized cells accumulate around the injury site, releasing significant pro‐inflammatory cytokines like tumor necrosis factor‐ α (TNF‐α), interleukin‐1β (IL‐1β), and interleukin‐12 (IL‐12).^21^ These cytokines attract an influx of inflammatory cells to the injury site, resulting in an excessive inflammatory response that hinders neural regeneration, thus worsening the overall prognosis.

Recently, there has been a growing emphasis on the role of lymphocytes, particularly CD8^+^ T cells and NK cells, and their downstream XCL1‐XCR1 axis in the context of TBI and SCI.^22^, ^23^ Research has revealed its potential to exert detrimental effects on both conditions, profoundly affecting recovery of motor function and specific cognitive functions. It is now widely recognized that CD8^+^ T cells contribute negatively to the outcomes through the granzyme/perforin pathway. However, a paradox arises, as CD4^+^ T cells, which also secrete Granzyme B (GrB^+^)/perforin, do not exhibit these detrimental effects after TBI.^22^

To better understand the mechanisms underlying the harmful effects of lymphocytes in TBI and SCI, we have formulated a hypothesis centered on the chemokine XCL1 secreted by lymphocytes and its receptor XCR1. These may play a significant role in acquired neurologic injuries. It should be noted that CD8^+^ T cells and NK cells predominantly secrete XCL1, with minimal secretion by CD4^+^ T cells. This distinction elucidates why CD8^+^ T and NK cells hurt the prognosis after TBI and SCI. Specifically, the significant secretion of XCL1 by these cells attracts dendritic cells expressing XCR1 receptors to the injury site. These dendritic cells release substantial amounts of IL‐12, a pro‐inflammatory cytokine that initiates Th1 cellular immune responses, leading to nerve damage and influencing the prognosis.

If our hypothesis proves to be accurate, the XCL1‐XCR1 axis could become a promising therapeutic target for patients with TBI and SCI. Blocking this axis can improve the prognosis of these patients. Therefore, this review aims to summarize data related to the role of the XCL1‐XCR1 axis and its upstream and downstream components in the CNS. By exploring their relevance to acquired CNS injuries and aseptic immunity, our objective was to gather evidence to validate this hypothesis. This hypothesis implies a novel therapeutic approach to treat TBI and SCI.

## 2 LITERATURE SEARCH STRATEGY

To facilitate our analysis, we performed extensive research using two major scholarly databases, PubMed and Web of Science. Our search parameters focused on the XCL1‐XCR1 axis, the T‐cell response, TBI, and SCI. We meticulously reviewed articles related to the pathophysiology of TBI and SCI and the inflammatory reaction that ensues after TBI and SCI. To ensure the precision and relevance of our findings, we meticulously weed out obsolete and repetitive studies.

## 3 PATHOPHYSIOLOGY OF TBI AND SCI

### 3.1 Pathophysiology of TBI

TBI occurs when the brain is damaged due to physical impact or penetrating head injury, causing the death of numerous brain cells and triggering various neuroinflammatory and pathophysiological responses.^24^ TBI has a high global morbidity and mortality rate^25^ and can result in a variety of neurological and cognitive deficits and, in severe cases, even death.^26^, ^27^, ^28^ Patients with TBI often experience long‐term consequences, such as developing neurodegenerative diseases like dementia or Parkinson's syndrome.^29^

TBI has an estimated prevalence of approximately 0.013% in China, which is consistent with reports from other countries.^30^ A study also estimates that there are around 770,060–890,990 new cases of TBI in China every year, with patient care costs ranging from 28,000 to 129,000 yuan.^31^ TBI causes significant physical suffering and imposes substantial economic burdens on patients. Thus, researchers must explore practical, safe, and cost‐effective treatments for TBI, given the current absence of clinically validated protocols.^32^, ^33^

TBI includes primary and secondary injuries.^34^ Primary injury disrupts the blood–brain barrier and causes the immune cells to aggregate in the injured area. Secondary injury can last from hours to months and cause significant brain damage, resulting in neurodegeneration, motor dysfunction, cognitive impairment, and comorbid neuropsychiatric disorders.^8^, ^35^

The pathophysiological mechanisms that underlie secondary injury comprise a range of processes, including glutamate excitotoxicity, mitochondrial dysfunction, oxidative stress, lipid peroxidation, neuroinflammation, axonal degeneration, and apoptotic cell death.^8^, ^29^, ^32^, ^36^, ^37^ Of these processes, the inflammatory response significantly impacts secondary damage. The inflammatory response of the CNS is an aseptic inflammatory response characterized by the overproduction of various cytokines and chemokines and the massive accumulation of different immune cells in the inflammatory area.^38^, ^39^

The CNS has an inflammatory response, which can accelerate tissue repair and protect against viral and bacterial invasion during the early stages of injury. However, extended activation of such aseptic inflammatory responses can have adverse consequences. This prolonged activation can exacerbate brain damage, impede recovery of neurological function, and ultimately result in significant neuronal necrosis and a poor prognosis.^40^, ^41^ Therefore, monitoring and managing the inflammatory response is crucial to ensure that it does not cause long‐term damage.^42^

### 3.2 Pathophysiology of SCI

SCI is a debilitating condition that causes degeneration of the spinal cord parenchyma due to direct or indirect external factors. This degeneration leads to impairment of the sensory, motor, and autonomic systems in an incapacitating state.^43^ Traumatic SCI is commonly associated with traffic accidents, falls, and violent acts. Conversely, nontraumatic SCI is primarily associated with degenerative cervical spondylosis.^44^ The outcome of SCI is a devastating state of sensory, motor, and autonomic dysfunction, which can severely affect the quality of life of those who experience it.

Approximately 2–3 million people worldwide are estimated to have SCI, with an annual increase of 250,000–500,000 new cases.^45^ In the United States, the annual incidence of SCI is approximately 54 cases per million people, translating to 17,730 new cases yearly, with an average annual medical cost of $76,327 per patient with SCI.^46^ These high treatment costs impose a significant financial burden and psychological stress on patients. Currently, effective treatments to promote functional recovery are lacking, and available treatments are limited to alleviating pain and infection symptoms during acute and chronic phases, as well as surgical interventions aimed at preventing further injury.^47^ This challenge mainly arises from an incomplete understanding of the complex mechanisms underlying SCI.

SCI is analogous to TBI in that it comprises two phases: primary injury and secondary injury. The first is caused by direct mechanical trauma to spinal cord tissue, resulting in demyelination, neuronal and axonal necrosis, and disruption of the blood–spinal cord barrier.^48^, ^49^ On the contrary, the latter involves a complex interplay of pathophysiological mechanisms, including local hemorrhage, ischemia, edema, ionic imbalances, oxidative stress, lipid peroxidation, and inflammatory responses.^49^, ^50^, ^51^, ^52^

The inflammatory process in SCI involves the activation of several cell types, including microglia, astrocytes, T cells, neutrophils, monocytes, and noncellular mediators. These cells significantly influence the pathological progression of acute and chronic SCI, with their beneficial or detrimental effects.^52^, ^53^, ^54^ Given that secondary injury is prolonged and leads to the accumulation and mobilization of more cells than primary injury, prioritizing secondary injury research is paramount in treating SCI.

## 4 THE ROLE OF THE XCL1‐XCR1 AXIS IN TBI AND SCI

### 4.1 Introduction to XCL1

XCL1 belongs to chemokines, which are a class of cytokines or signaling proteins secreted by various types of cells. These molecules, characterized by a low molecular weight ranging from 8 to 12 kDa and a conserved structural pattern, play a crucial role in immune cell chemotaxis, the initiation of inflammatory responses, and the exacerbation of neural damage when they bind to their respective receptors.^55^, ^56^ Chemokines have four subgroups based on the number and conformation of highly conserved cysteine residues in their amino acid sequences: CXC (α chemokines), CC (β chemokines), XC (γ chemokines), and CX3C (δ chemokines).^57^ Furthermore, their receptors are organized into four subfamilies: CXCR, CCR, XCR, and CX3CR.^58^ Chemokines that bind to seven transmembrane receptors are coupled to heterotrimeric G proteins, commonly known as G‐protein‐coupled receptors (GPCR). Upon binding, conformational changes are triggered, leading to intracellular signaling cascades that promote cell activation, aggregation, and secretion.

The role of chemokines in facilitating cell migration and secretion during inflammatory responses and autoimmune diseases is widely recognized.^59^, ^60^ Recent studies have highlighted the importance of chemokines in the physiologic and pathologic processes of the nervous system.^56^ These processes promote neuronal development, exacerbate neuroinflammation, and regulate the release of synaptic transmitters. Furthermore, the association between chemokines and various neurological disorders, including multiple sclerosis, Parkinson's disease, Huntington's disease, and Alzheimer's disease,^61^, ^62^, ^63^, ^64^, ^65^ has been extensively reported. In particular, specific chemokines such as CCL2, CCL3, CCL4, CCL9, CCL11, CX3CL1, and CXCL5 have shown significant changes after TBI and SCI.^66^, ^67^, ^68^ The altered expression of these chemokines after TBI and SCI suggests their potential as biomarkers to assess the extent of injury and as therapeutic targets for intervention.

In the mouse genome, there are four subgroups of chemokines, one of which is XC, which consists of only one member, XCL1. XCL1 is also known as lymphokines, single C motif‐1 (SCM‐1), or activation‐induced T‐cell‐derived and chemokine‐associated molecule (A‐TAC). Various immune cells, including activated CD8^+^ T cells, NK cells, NKT cells, CD4^+^ T cells, γδ‐T cells, and thymic medullary epithelial cells, can express XCL1.^69^ However, activated CD8^+^ T cells and NK cells are the primary producers of this chemokine.

The gene encoding XCL1 is present in several mammals, including humans and mice. Although the XC‐like chemokine of mice comprises only XCL1, the human genome harbors two highly homologous XC‐like chemokine genes: XCL1 and XCL2, also known as SCYC1 and SCYC2, respectively. Previous research indicates that XCL1 transcripts are abundant in tissues such as the spleen, thymus, intestine, and peripheral blood leukocytes.^69^ However, recent studies have expanded the expression of XCL1 in the cortical, thalamic, and hippocampal regions of the brain, as well as in the trigeminal nerve. In particular, its upregulation following injury has also been observed.^70^, ^71^ These findings lay the foundation for exploring the functional role of XCL1 in the context of TBI and SCI.

### 4.2 Introduction to XCR1

XCR1 is a prototypical G‐protein‐coupled receptor that comprises seven transmembrane domains. It functions as the primary receptor for XCL1 and is predominantly expressed in dendritic cells (DC). Upon interaction with XCL1, cells expressing XCR1 exhibit calcium mobilization and chemotactic responses.^72^ In mice, classic type 1 dendritic cells, including CD8α^+^ cDCs in lymphoid tissues and CD103^+^ CD11b^−^ cDCs in non‐lymphoid soft tissues, can migrate to draining lymph nodes after activation and maturation. In humans, CD8α^+^ cDC1 corresponds to CD141^+^ cDCs, and cDC1 expresses specifically the chemokine receptor XCR1.^73^, ^74^ CD8^+^ and CD103^+^ CD11b^−^ DCs in mice are vital in efficiently processing cell‐associated antigens and presenting them to CD8^+^ T cells.^72^, ^73^ Subsequently, CD8^+^ T cells can activate cytotoxic T lymphocytes, which participate in various sterile immune and inflammatory responses within the nervous system. Similarly, human CD141^+^ dendritic cells effectively present soluble and cell‐associated antigens to CD8^+^ T cells.^73^

It is important to note that XCR1 is expressed explicitly in dendritic cells, which are highly specific in the presentation of antigens to lymphocytes. Consequently, XCR1 could be a valuable marker for future dendritic cell experiments. Furthermore, it is essential to mention that the effects of XCL1 become particularly prominent upon binding to XCR1.^75^ Dendritic cell subpopulations are the primary cell subtype expressing XCR1,^73^ and, as a result, the interactions between XCL1 and XCR1 significantly impact immune responses mediated by lymphocytes and dendritic cells.

### 4.3 Pro‐inflammatory effect of XCL1‐XCR1 axis

Examination of the expression of XCL1 and XCR1 after TBI revealed an increase.^71^ Previous research indicated that the expression of XCL1 and XCR1 also increased in a mouse model of type 1 diabetes, specifically the streptozotocin model.^76^ It is important to note that the study primarily demonstrated the presence of XCR1 in neuronal cell bodies in the CNS, such as the lumbar spine, without providing detailed information on the precise location of cell bodies containing XCR1. Another study reported increased XCR1 expression near the injury site in the trigeminal nerve. The authors suggest that the XCL1‐XCR1 axis could be crucial in the peripheral and central pain pathways of the trigeminal nerve. They also suggested that blocking the XCL1‐XCR1 axis could hold promise for alleviating pain in the orofacial region.^70^ Recent research has shown that neutralizing antibodies targeting XCL1 and ITGA9 can effectively prevent disease progression in experimental encephalomyelitis.^77^ These findings underscore the importance of the XCL1‐XCR1 axis as an essential signaling pair for neuroinflammatory diseases, including TBI and SCI.

Numerous studies have extensively investigated the role of XCL1 and XCR1 in inflammatory diseases. In particular, the XCL1‐XCR1 axis plays a significant role in the pathogenesis of rheumatoid arthritis by inducing Th1‐mediated pro‐inflammatory responses. In patients with rheumatoid arthritis, XCL1 manifests itself in the synovium, while XCR1 is a characteristic feature of infiltrating mononuclear and synovial cells.^78^ Furthermore, increased expression of XCL1 has been observed in tissues affected by Crohn's disease and in inflammatory responses of diabetic mice.^79^, ^80^ These findings collectively suggest that the expression of XCL1 and XCR1 increases in various sterile inflammatory diseases in humans and mice. Therefore, we hypothesize that the XCL1‐XCR1 axis plays a pro‐inflammatory role in secondary injury after TBI and SCI.

Given that aseptic inflammation is a crucial component of secondary injury in these conditions. Upregulation of XCL1 expression in various sterile inflammatory diseases highlights the potential of the XCL1‐XCR1 axis as a promising therapeutic target to mitigate neuroinflammation. This axis can contribute to protective and pathological inflammatory responses.^69^ Therefore, inhibiting the XCL1‐XCR1 axis may provide the key to alleviating excessive inflammatory responses in sterile neuroinflammatory diseases such as TBI and SCI. On the contrary, activating the XCL1‐XCR1 axis may trigger an enhancement of neuroinflammation, such as the pro‐inflammatory reactions of helper T1 cells (Th1 cells) and helper T17 cells (Th17 cells).

Chemokines and their receptors are crucial in various cellular processes, including the immune response and inflammation. A deeper understanding of the molecular mechanisms of chemokine signaling can offer novel insights into developing therapeutic strategies for diseases such as TBI and SCI.

## 5 THE ROLE OF T CELL RESPONSE IN TBI AND SCI

Following nerve injury, lymphocytes migrate to the injury site within 24 h and aggregate between 3 and 5 days.^81^ CD8^+^ T cells, a component of the upstream pathways that secrete XCL1, play a crucial role in the CNS. These lymphocytes are actively involved in the pathogenesis of TBI. Both clinical observations in patients with TBI and the animal models of TBI have reported an accumulation of immune cells within the CNS.^82^, ^83^ This accumulation follows a specific timeline, with neutrophils being the first to migrate to the brain injury site within 24 h after TBI. Subsequently, within 3–5 days, macrophages, lymphocytes, and dendritic cells accumulate in the injury area. These cells release a substantial amount of pro‐inflammatory cytokines and chemokines, perpetuating the inflammatory response and causing damage to neuronal tissue.^81^

Previous studies have suggested that mice with knockout T or B cells show better outcomes after SCI.^84^ This finding implies that accumulation of lymphocytes after injury can have harmful effects. Furthermore, CD8^+^ T cells have been identified to be capable of activating apoptotic programs by producing pro‐inflammatory cytokines and lysozymes within the brain.^85^, ^86^ In a rat model of cerebral ischemia, rats depleted of CD8^+^ T cells showed smaller infarct volumes than wild‐type rats.^87^ A study examining the impact of CD8^+^ T cells in TBI revealed the persistence of cytotoxic lymphocytes in the brain long after the injury, resulting in progressive neurological and motor function deterioration.

Conversely, mice with genetically or pharmacologically deficient CD8^+^ T cells demonstrated neurological improvement and reduced myelin damage in the spinal cord within 8 weeks.^22^ A separate TBI study found that CD8^+^ T cells infiltrated the brain, promoted by astrocyte‐derived IL‐15, and secreted GrB early at 24 h, initiating the caspase‐3/poly ADP ribose polymerase (PARP) pathway and subsequent neuronal apoptosis.^88^ Moreover, critical experiments conducted in perforin knockout mice revealed that CD8^+^ T cell‐induced neurotoxicity extends beyond the release of perforin. It involves the mediation of Th1 pro‐inflammatory cytokine responses 1 week after ischemic stroke.^89^ Considering these findings and the ability of the XCL1‐XCR1 axis to activate Th1 pro‐inflammatory cytokine responses, it is reasonable to assume that CD8^+^ T cells may activate Th1 pro‐inflammatory cytokine responses through their downstream XCL1‐XCR1 axis.

Following SCI, the physiological functioning of the spinal cord and interactions among various cell types are disrupted, significantly impacting the recovery process.^90^, ^91^ Recent studies have increasingly emphasized the role of lymphocytes in secondary damage associated with SCI. For instance, research by Liu et al.^92^ has indicated that perforin, primarily produced by CD8^+^ T cells, exacerbates the disruption of the blood–spinal barrier and intensifies the inflammatory response. This finding suggests that lymphocytes, especially CD8^+^ T cells, contribute negatively to SCI.

Moreover, specific lymphocytes, including CD8^+^ T cells and NK cells, release XCL1, while some dendritic cells express XCR1 receptors. Consequently, it is reasonable to hypothesize that these lymphocytes may exacerbate secondary injury by releasing GrB and activating the XCL1‐XCR1 axis. This activation triggers a cascade of downstream inflammatory responses that impede recovery after SCI and TBI.

In summary, these findings indicate that lymphocytes, particularly CD8^+^ T cells, have a detrimental effect on TBI and SCI. We should consider their role in secondary injury when developing therapeutic interventions for SCI and TBI. Further research is needed to understand better the mechanisms underlying lymphocyte‐mediated secondary injury, which may lead to the development of more effective treatments for these devastating conditions.

## 6 THE ROLE OF UPSTREAM AND DOWNSTREAM PATHWAYS OF THE XCL1‐XCR1 AXIS IN TBI AND SCI

Notably, several studies have demonstrated an increase in the expression of XCL1 and XCR1 after TBI and trigeminal nerve injury.^70^, ^71^ Although this pathway has not yet been examined in the context of SCI, it is plausible that a similar pathway exists within the CNS. After SCI, there is a rapid surge in interleukin‐15 (IL‐15) within 24 h of injury, which can attract CD8^+^ T cells and NK cells to the spinal cord injury site. These cells release XCL1 and interferon‐gamma (IFN‐γ), leading to cascading responses.

The research on XCL1 and its therapeutic potential for various immune and aseptic inflammatory diseases is nascent. Nevertheless, there is growing evidence of its potential therapeutic relevance. IL‐15 has been used clinically in tumor therapy, where it recruits CD8^+^ T cells and NK cells to the tumor site, leading to the secretion of XCL1 and the subsequent attraction of dendritic cells expressing XCR1 receptors. This cascade of events inhibits tumor growth and metastasis.^66^

However, in contrast to its application in tumor therapy, this pathway appears to play a harmful role in TBI and SCI by exacerbating the inflammatory response in the secondary injury phase. Over time, the downstream components activate, creating a positive feedback loop that sustains the inflammatory response, damages the nervous system, and affects the prognosis. If the hypothesis regarding such a pathway in the CNS after injury holds, interventions targeting any segment of this pathway could potentially lead to significant improvements in the prognosis of neurological injuries.

As mentioned, T lymphocytes and the downstream XCL1‐XCR1 axis have a detrimental effect on aseptic immune responses after TBI and SCI. However, this pathway encompasses multiple other components. In the subsequent sections, we will systematically review these components that respond to CNS injury. We will follow a chronological order and a top‐down path, beginning with the initial substances found after the injury.

### 6.1 The XCL1‐XCR1 axis experiences changes in its upstream and downstream pathways after TBI AND SCI

Following injury, IL‐15 emerges as an early responder, closely associated with CD8^+^ T cells. As a member of the 4‐α‐helix bundle glycoproteins family, IL‐15 is critical in the activation, proliferation, activation, and survival of T and NK cells.^93^, ^94^, ^95^ The clinical significance of IL‐15 extends to several sterile inflammatory diseases, including celiac disease, rheumatoid arthritis, inflammatory synovitis, and certain inflammatory bowel diseases.^96^, ^97^, ^98^, ^99^

In TBI and SCI, experiments have demonstrated that IL‐15 is overexpressed in the cortex and cerebrospinal fluid, peaking within 24 h of the injury. This overexpression can be attributed to astrocytes, which produce most IL‐15, with a smaller fraction originating from microglia.^88^, ^100^, ^101^, ^102^ Of note, astrocyte‐secreted IL‐15 exacerbates the situation^103^ by promoting the aggregation, activation, and potentiation of CD8^+^ T cells and NK cells at the injury site.^102^

The experimental evidence shows that IL‐15 significantly regulates the initial inflammatory response following nerve injury. Studies on chronic constriction injury (CCI) of the sciatic nerve and TBI reveal that IL‐15 is crucial in T‐cell recruitment and activation at the injury site. Injecting recombinant IL‐15 (rIL‐15) into rats with TBI resulted in increased T‐cell infiltration into the cerebral cortex, elevated GrB expression, and neuronal apoptosis, highlighting the pro‐inflammatory effects of IL‐15 in the CNS.^88^ On the other hand, specific antibodies that reduced IL‐15 activity significantly reduced T‐cell infiltration after CCI and inhibited pro‐inflammatory responses.^92^ These findings further support the idea that IL‐15 contributes to the inflammatory response in neurological injuries. Increased IL‐15 expression may attract CD8^+^ T cells, NK cells, and other lymphocytes to the site of inflammation. As a result, interventions to reduce IL‐15 activity could be a promising strategy to mitigate inflammation and improve outcomes in TBI and SCI.

Lymphocytes are recognized for their ability to reach the site of a nerve injury within a day and for their propensity to aggregate between 3 and 5 days after that.^81^ Upon the accumulation of CD8^+^ T cells and NK cells at the injury site, a substantial amount of XCL1 is released. Remarkably, a significant increase of XCL1 in the brain is observed approximately 4–7 days after TBI. As such, it is postulated that astrocytes first release a considerable amount of IL‐15 after SCI, which attracts CD8^+^ T cells and NK cells to the injury site. In turn, these lymphocytes release substantial amounts of XCL1 that induce dendritic cells expressing XCR1 receptors to migrate to the injury site. Dendritic cells may release IL‐12, a crucial cytokine responsible for transforming the differentiation of Th0 CD4^+^ T cells into Th1 and Th17 cells. Moreover, these Th1 and Th17 cells release large amounts of interleukin‐1 (IL‐1) and interleukin‐17 (IL‐17), significantly amplifying pro‐inflammatory cytokine responses, thus contributing to sustained nerve damage in TBI and SCI, ultimately affecting the prognosis. Th1 and Th17 cells have been documented to exacerbate pro‐inflammatory cytokine responses in neurological injuries.^89^

According to recent studies, XCR1 displays a significant elevation, slightly preceding the upsurge of XCL1 in the brain at 24 h after TBI.^71^ This phenomenon may be attributed to dendritic cells with XCR1 receptors that accumulate at the injury site without necessitating XCL1 chemotaxis. In other words, XCL1 can activate more dendritic cells expressing XCR1 chemotactically to reach the injury site, but this activation is not mandatory. Even in the absence of XCL1, some XCR1‐expressing dendritic cells can accumulate at the injury site and stimulate IL‐12 secretion flowing SCI, elucidating the rapid increase in IL‐12 expression after TBI and SCI, as presented in Table 1.

**TABLE 1:** Changes in the upstream and downstream pathways of the XCL1‐XCR1 Axis in traumatic brain injury and spinal cord injury.

Lymphocyte/Immune molecules	Time points	Source	Species	Diseases	Outcome	References
IL‐15	6 h ↑	Astrocytes Microglia	Adult male Wistar rats	TBI	Promotes the aggregation, value‐added and activation of CD8^+^ T cells and NK cells	[88, 101, 102]
CD8^+^ T cell	7 day ↑	N/A	Female C57BL/6 mice	SCI	Secret XCL1 and INF‐γ	[104]
NK cell	1 day ↑	N/A	Male C57BL/6 mice	TBI	Secret XCL1 and INF‐γ	[83, 105]
IFN‐γ	1 day ↑	CD8^+^T cell NK cell Th1 cell Th17 cell	Female Wistar rats	SCI	Intensify inflammatory response, promotes the aggregation and activation of DC cell	[75, 106]
XCL1	4 day ↑	CD8+T cell NK cell	Male C57BL/6J mice	TBI	Chemotaxis, aggregation of DC cells with XCR1 receptor	[69, 71]
XCR1+ DC cell	1 day ↑	N/A	Male C57BL/6J mice	TBI	Present antigens and activate lymphocytes, secret IL‐12	[71, 75]

*Note*: ↑, increase.

Abbreviations: DC, dendritic cell; IFN‐γ, interferon‐gamma; IL‐12, interleukin‐12; IL‐15, interleukin‐15; N/A, not available; SCI, spinal cord injury; TBI, traumatic brain injury; Th1 cells, helper T1 cells; Th17 cells, helper T17 cells.

### 6.2 Pro‐inflammatory effect of the upstream and downstream pathways of the XCL1‐XCR1 axis after TBI and SCI

The precise roles of IL‐15, CD8^+^ T cells, and NK cells in the pathogenesis of TBI and SCI remain unclear. However, it is speculated that IL‐15 promotes the aggregation and activation of CD8^+^ T cells and NK cells at the injury site. Subsequently, these cells secrete significant amounts of XCL1 and INF‐γ.^104^, ^105^, ^106^ XCL1 then activates many dendritic cells, primarily CD8α^+^and CD103^+^ dendritic cells containing XCR1 receptors. These dendritic cells, in turn, secrete substantial amounts of IL‐12 since dendritic cells are the primary source of IL‐12. This IL‐12 and INF‐γ drive Th0 cell differentiation into Th1 and Th17 cells, a well‐established process in various human body tissues and clinical tumor treatment.^75^

The evidence that IL‐15 can chemotactically attract CD8^+^ T cells and NK cells and that mice with knockout CD8^+^ T cells and NK cells exhibit better motor recovery after neurological injury^22^ supports the notion that these cells may play a detrimental role in the inflammatory response after TBI and SCI. These interventions aimed at blocking specific components of this pathway could reduce the inflammatory response and offer neuroprotection.

The observed differences between CD8^+^ T cells and CD4^+^ T cells and their respective roles provide valuable information. CD4^+^ T cells do not secrete XCL1, essential to trigger Th1 and Th17 inflammatory responses. Therefore, eliminating CD4^+^ T cells did not significantly improve the prognosis of neurological injury.^22^ This finding indirectly supports the hypothesis that the XCL1‐XCR1 axis and the downstream inflammatory responses play a role in secondary injury after TBI and SCI.

**FIGURE 1:**
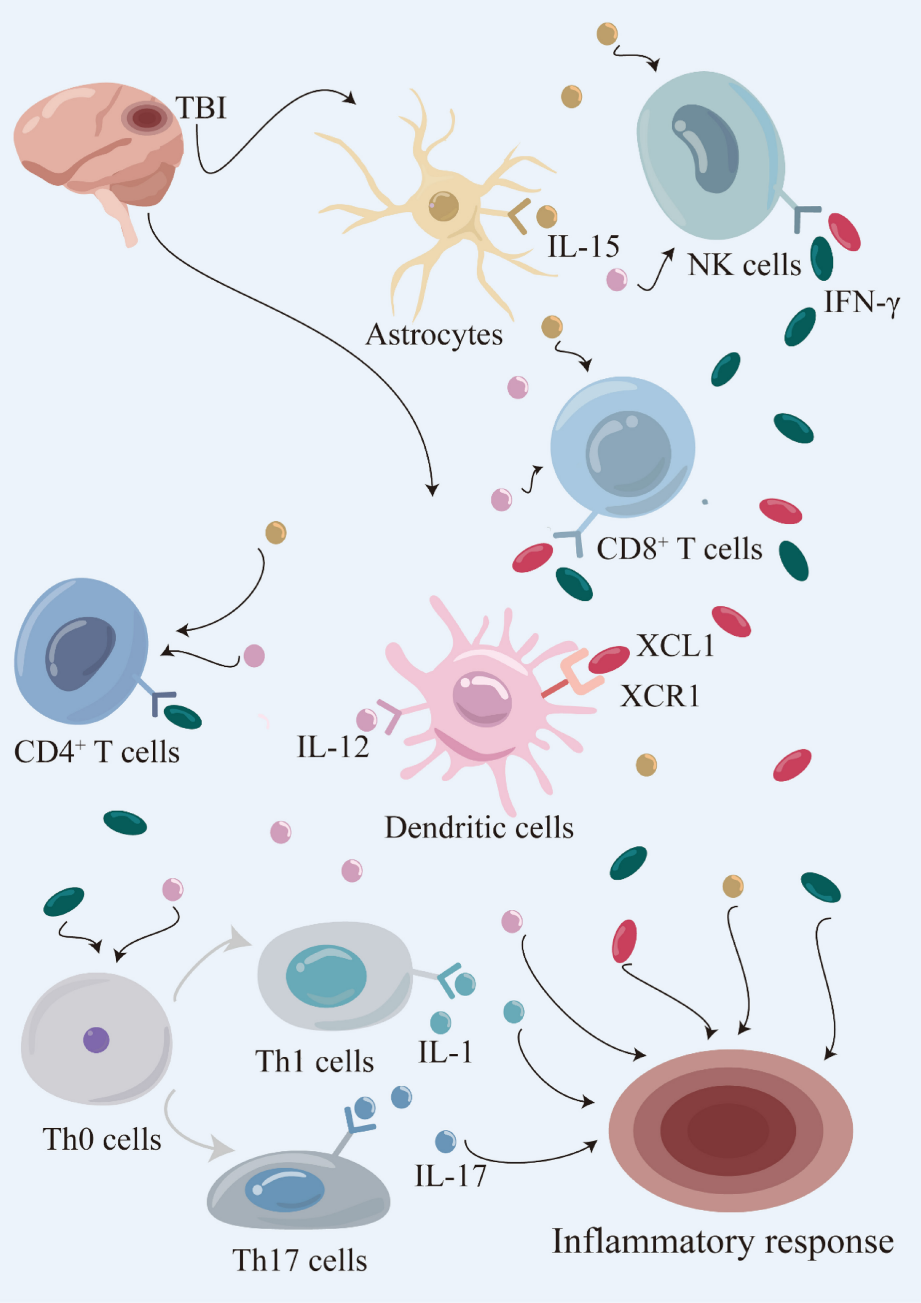
The changes in various inflammatory cells and molecules within the XCL1‐XCR1 pathway after traumatic brain injury (TBI): Astrocytes and dendritic cells increase and secrete IL‐15 and IL‐12, respectively. As a result, IL‐15 and IL‐12 activate and aggregate downstream CD8^+^ T cells, CD4^+^ T cells, and NK cells. These cells, in turn, release chemokines XCL1 and IFN‐γ, which trigger further inflammatory responses. Moreover, XCL1 selectively binds to dendritic cells with XCR1 receptors, and these dendritic cells, in response, can release more IL‐12. Finally, IL‐12 and IFN‐γ trigger Th0 cell differentiation into Th1 and Th17 cells, leading to increased secretion of IL‐1 and IL‐17. These inflammatory factors worsen the inflammatory response, posing significant damage during the acute and chronic phases.

**FIGURE 2:**
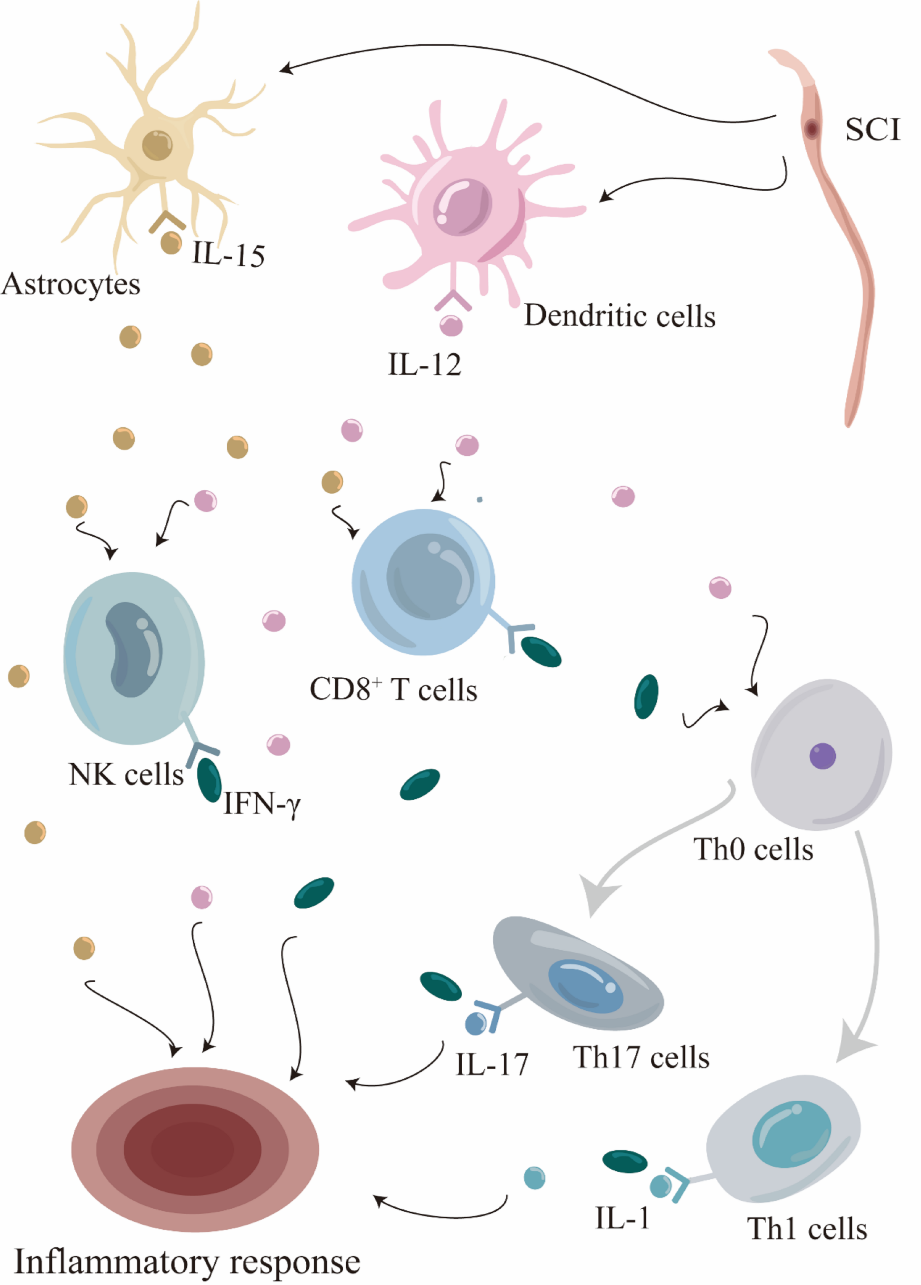
The alterations in inflammatory cellular and molecular profiles after spinal cord injury share similarities with those observed after traumatic brain injury. Initially, astrocytes and dendritic cells release substantial amounts of IL‐15 and IL‐12, respectively. These cytokines collectively activate downstream CD8^+^ T cells and NK cells, both of which will secrete IFN‐γ, with CD8^+^ T cells being the main contributors. Subsequently, the collaborative action of IL‐12 and IFN‐γ triggers the differentiation of Th0 cells into Th1 and Th17 subsets, leading to an increased secretion of inflammatory factors such as IFN‐γ, IL‐1, and IL‐17, thus exacerbating the inflammatory cascade. However, the current literature lacks comprehensive research on the expression dynamics of the XCL1‐XCR1 axis after spinal cord injury, highlighting a promising avenue for future research endeavors.

Although the specific details of this pathway in the context of CNS injury are not fully understood, it is essential to note that the pro‐inflammatory response of particular cytokines, known as Th1 and Th17 cytokines, can worsen nerve damage caused by inflammation.^21^, ^107^ This finding leads us to believe that a pathway involving various components such as IL‐15, IL‐12, CD8^+^ T cell, CD4^+^ T cell, NK cell, XCL1, XCR1^+^ dendritic cell, IFN‐γ, Th0 cells, Th1 cells, and Th17 cell may have a pro‐inflammatory effect after TBI and SCI. Blocking any part of this pathway could have positive effects and ultimately improve the prognosis of neurological injuries, making it a promising therapeutic approach (Figures 1 and 2).

## 7 CONCLUSIONS AND PERSPECTIVE

The current research on the XCL1‐XCR1 axis in immunoinflammatory damage in neurological diseases is still in its nascent stages. However, compelling evidence suggests that this axis and its associated pathways are pivotal in the CNS. This area presents a promising avenue for future research with a high degree of innovation and potential clinical applicability. Further research will provide valuable insights and pave the way for potential clinical applications in treating TBI and SCI.

If the hypothesis on the existence of the proposed pathway in TB and SCI is confirmed, interventions aimed at targeting any part of this pathway could hold significant promise for improving the prognosis of patients. The aseptic inflammatory response often causes more damage than the primary injury itself, so blocking this pathway to mitigate the inflammatory response could represent an attractive clinical treatment option. Neutralizing IL‐15 antibodies seems to be a feasible starting point for reducing elevated levels of IL‐15 based on substantial evidence. Further basic research and clinical investigations can help validate the effectiveness of these interventions and their effects in enhancing recovery and outcomes in patients with TBI and SCI.

**FIGURE 3:**
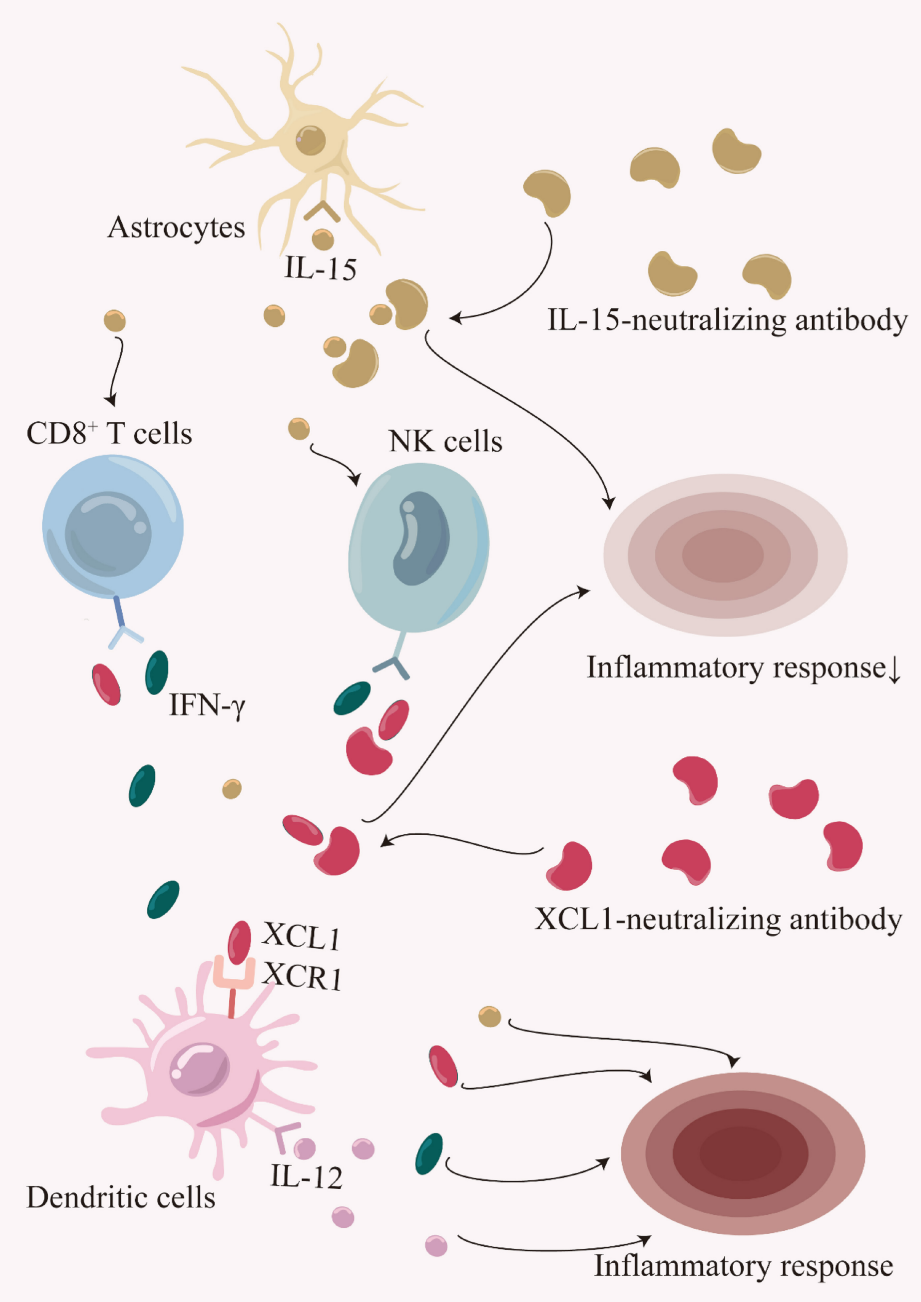
The content posits that introducing inhibitors at any point within the XCL1‐XCR1 inflammatory pathway has the potential to impede a cascade of downstream inflammatory molecules. This disruption can be expediently executed through the utilization of common IL‐15‐neutralizing and IL‐12‐neutralizing antibodies. For instance, IL‐15‐neutralizing antibodies can hinder the chemotactic influence of IL‐15 on CD8^+^ T and NK cells, thereby reducing CD8^+^ T and NK cells that exacerbate the inflammatory responses and alleviate the overall inflammatory reactions. Similarly, XCL1‐neutralizing antibodies can inhibit the specific binding between the chemoattractant molecule XCL1 and its receptor XCR1, thereby reducing the chemoattractant effect of XCL1 in dendritic cells with XCR1 receptors. Consequently, the diminished attraction reduces dendritic cells and IL‐12, ultimately alleviating the inflammatory response.

Although no studies have yet examined whether blocking the XCL1‐XCR1 axis can reduce the inflammatory response and offer neuroprotection after TBI and SCI, the upregulation of XCL1 and XCR1 after neurological injury suggests that blocking this axis with neutralizing antibodies against XCL1 could be an effective strategy to reduce the inflammatory response. Using XCL1‐neutralizing antibodies to prevent the recruitment of dendritic cells with XCR1 receptors and then reducing Th0 cell conversion to pro‐inflammatory Th1 and Th17 cells by reducing DC‐produced IL‐12 could alleviate the neuroinflammatory response (Figure 3). In theory, apart from the two neutralizing antibodies mentioned above, the incorporation of inhibitors at any juncture along the pro‐inflammatory pathway has the potential to exert anti‐inflammatory effects. Specifically, antagonists that aim to inhibit dendritic cell activation, CD8^+^ T cells, and NK cells, or the toxic effects of IL‐12, IL‐1, IL‐17, and IFN‐γ, provided they effectively decrease the quantity or activity of these inflammatory cells and cytokines, can mitigate the inflammatory response within the pro‐inflammatory pathway. Consequently, the XCL1‐XCR1 axis, along with its upstream and downstream pathways, presents numerous potential therapeutic targets. These proposed interventions promise to improve the prognosis of neurological injuries and warrant further investigation to assess their effectiveness in preclinical and clinical settings.

## AUTHOR CONTRIBUTIONS

Each author is expected to have made substantial contributions to the conception. Tian Cheng, Hongjian Liu, and Chao Jiang designed the work. Mingkang Zhang, Xiaonan Han, Liyan Yan, Yikun Fu, and Junmin Wang wrote the manuscript. Hongwei Kou and Chunfeng Shang created the figure of the manuscript. Jian Wang intellectually contributed and revised the manuscript. All the authors read and approved the manuscript.

## FUNDING INFORMATION

This work was supported by the Henan Province High‐Level Talent Internationalization Training Funding Project (Yuke(2022)‐3) and the National Natural Science Foundation of China (82371339).

## CONFLICT OF INTEREST STATEMENT

The authors declare that they have no conflict of interest.

## Data Availability

This is a review; therefore, availability of data and materials is not applicable.
